# Plasmablastic Lymphoma: A Systematic Review

**DOI:** 10.1100/tsw.2011.59

**Published:** 2011-03-22

**Authors:** Jorge J. Castillo, John L. Reagan

**Affiliations:** The Warren Alpert Medical School of Brown University, Division of Hematology and Oncology, The Miriam Hospital, Providence, RI, USA

**Keywords:** plasmablastic lymphoma, PBL, HIV, AIDS, chemotherapy

## Abstract

Plasmablastic lymphoma (PBL) is a very aggressive variant of diffuse large B-cell lymphoma initially described in the oral cavity of HIV-infected individuals. PBL represents a diagnostic challenge given its characteristic morphology and lack of CD20 expression, and also a therapeutic challenge, with early responses to therapy, but with high relapse rates and poor prognosis. In recent years, our understanding and clinical experience with PBL has increased in both HIV-positive and -negative settings. However, given its rarity, most of the data available rely on case reports and case series. The main goal of this article is to systematically review the most recent advances in epidemiology; pathophysiology; clinical, pathologic, and molecular characteristics; therapy; and prognosis in patients with PBL. Specific covered topics include new pathological markers for diagnosis, its association with Epstein-Barr virus, and the need of more intensive therapies.

### INTRODUCTION

Plasmablastic lymphoma (PBL) is a relatively new clinical entity described as a distinct subtype of diffuse large B-cell lymphoma (DLBCL), characterized by its aggressive nature and plasmacytic differentiation[1]. In the original report, 15 out 16 patients were infected with HIV and all the patients had involvement of the oral cavity[2]. In the last decade, several case reports and series have been published, accounting for no more than 250 cases[3,4]. More recently, however, several cases of PBL involving extraoral sites have been reported in immunocompetent individuals[5,6,7,8,9].

PBL remains a diagnostic challenge given its peculiar morphology and an immunohistochemical profile similar to plasma cell myeloma (PCM). Furthermore, PBL is a therapeutic challenge with a clinical course characterized by a high rate of relapse and death. It is important to note that there is no established standard of care for PBL as it is unknown if the outcome of patients with PBL has improved in the highly active antiretroviral therapy (HAART) era. However, as a better understanding of the pathophysiology and biology develops, different options are of potential interest for the treatment of PBL.

The purpose of this review is to summarize, in a comprehensive but concise manner, the existing data regarding epidemiology, pathogenesis, clinical and pathologic features, diagnostic criteria, therapy, and prognostic factors on patients with PBL. Plasmablastic microlymphomas, also known as large B-cell lymphomas arising from human herpesvirus 8 (HHV8)–associated multicentric Castleman disease[10], are a distinct subtype of lymphoma and are beyond the scope of this review.

### EPIDEMIOLOGY

PBL is a rare entity, thought to account for approximately 2.6% of all AIDS-related lymphomas (ARLs)[11], although the exact rate of incidence is not known. Furthermore, the actual incidence of PBL not associated with HIV infection has not yet been determined. In the largest literature review of 228 patients with PBL, 157 patients (69%) were HIV-positive and 71 (31%) were HIV-negative[5]. In HIV-negative patients, approximately a third of the patients have some form of iatrogenic immunosuppression, most often solid organ transplantation[4]. The remainder of the HIV-negative patients is apparently immunocompetent; in a recent case series from Korea, none of the patients reported showed evidence of immunosuppression[7].

The majority of patients with PBL are men, particularly the HIV-positive cases, with a mean age at presentation of 39 years in HIV-positive patients and 58 years in HIV-negative patients[3]. PBL is an even rarer phenomenon in children, with only five case reports in the literature[12,13,14,15,16]. Due to the current scarcity of data, it is unclear if there is a racial or ethnic predominance in PBL patients. However, cases have been reported in populations from different continents, such as Europe[6,17,18,19,20,21,22], Asia[7,16,23,24,25,26,27], Africa[28,29], Australia[30,31], South America[32,33], and North America[9,34,35,36,37,38,39].

### PATHOGENESIS

The pathogenesis of PBL is poorly understood and likely dependent on a variety of molecular events and pathways. Based on immunohistochemical, molecular, and genetic studies, PBL is thought to derive from postgerminal-center, terminally differentiated, activated B cells, probably in transition from immunoblast to plasma cell. By definition, these cells have undergone class switching and somatic hypermutation; however, there are chromosomal aberrations in these processes likely associated with the development of malignancy. A recent study has shown recurring rearrangements involving MYC, a well-known oncogene, and the immunoglobulin gene[22]. In a sense, this is similar to what is seen in patients with Burkitt lymphoma, although the majority of these cases are thought to generate from germinal-center B-cells.

Similar to other ARLs, such as Burkitt, immunoblastic, and primary effusion lymphoma (PEL), PBL has a strong association with Epstein-Barr virus (EBV) infection. In a recent review, Carbone and colleagues cite a potential role of EBV infection as a B-cell protector from apoptosis connected to MYC overexpression[40]. In HIV-associated PBL, 74% of the cases showed presence of EBV within the tumor cells[3]. EBV infection has been demonstrated based on the expression of EBV-encoded RNA (EBER)[5]. EBER is a viral transcript found among the three different EBV latency patterns seen in lymphomas, making it a reliable marker for determining latent EBV infection.

The association between PBL and HHV8 at this time is unclear. Few studies have demonstrated expression of HHV8-associated proteins in PBL[41,42]; however, other studies do not support such an association[9,43]. Furthermore, it is unclear if these HHV8-associated PBL cases originated from multicentric Castleman disease, which should placed them in a different category[10].

## PATHOLOGIC FEATURES

### Morphology

PBL is characterized by a monomorphic proliferation of round- to oval-shaped cells with plasmacytoid features (i.e., abundant cytoplasm, eccentric nucleus, and a prominent central nucleolus)[1]. A perinuclear hof is sometimes seen. The background infiltrate contains small mature lymphocytes and may include apoptotic bodies, mitotic figures, and tingible-body macrophages, which impart a “starry-sky” appearance[1]. A plasmablastic morphology, however, may be seen in other lymphoproliferative disorders, such as plasmablastic PCM, Burkitt lymphoma, DLBCL with plasmacytoid differentiation, PEL, and anaplastic lymphoma kinase (ALK)–positive DLBCL.

### Immunophenotype

Advances in immunophenotyping have helped greatly when differentiating PBL from other neoplasms (Table 1). The hallmark immunohistochemical staining pattern of PBL is that of terminally differentiated B lymphocytes. PBL demonstrates little to no expression of leukocyte common antigen (CD45) or the B-cell markers CD20, CD79a, and PAX5. However, the plasma cell markers VS38c, CD38, multiple myeloma oncogene-1 (MUM1), and CD138 (syndecan-1) seem to be almost universally expressed (Fig. 1)[3,44]. There seems to be, however, some distinction of immunohistochemical patterns in patients with PBL based on their HIV status. In a literature review, HIV-positive patients showed a greater expression of CD20 and CD56 compared to HIV-negative patients[5]; however, the significance of this finding remains uncertain and could be due to misclassification.

**TABLE 1**
**Differential Pathological Diagnosis of PBL**

| Lymphoma Type | Burkitt | DLBCL | PBL | PEL | ALK + DLBCL | PCM |
|---|---|---|---|---|---|---|
| Association with HIV | ++ | ++ | +++ | +++ | - | - |
| Seen in immunocompetent patients | ++ | +++ | +/- | +/- | + | ++ |
| CD45 | + | + | +/- | +/- | +/- | + |
| CD20 | + | + | - | +/- | - | - |
| Plasma cell markers | +/- | +/- | + | + | + | + |
| Ki67 | High >90% | High <90% | High >60% | High >80% | High >80% | Low |
| EBER | + (60%) | +/- | + (80%) | + (90%) | - | - |
| Other | CD10+ | | BLIMP1+ | HHV8+ | ALK+ | Serum M-spike CRAB |

CRAB: hypercalcemia, kidney disease, anemia, and bone lytic lesions.

Recently, new methods for reliably identifying PBL have been developed. Positive regulatory domain 1 (PRDM1/BLIMP1) protein and activated transcription factor X-box binding protein 1 (XBP1) are proteins involved in terminal B-lymphocyte differentiation[45]. A recent study has shown that negative or weak staining for PAX5 and CD20 coupled with positive staining for PRDM1/BLIMP1 and XBP1 reliably identifies cases of PBL[21]. DLBCL, in contrast, exhibited the above staining pattern in <5% of the cases. However, in less developed countries, the pathological diagnosis of PBL could pose a challenge.

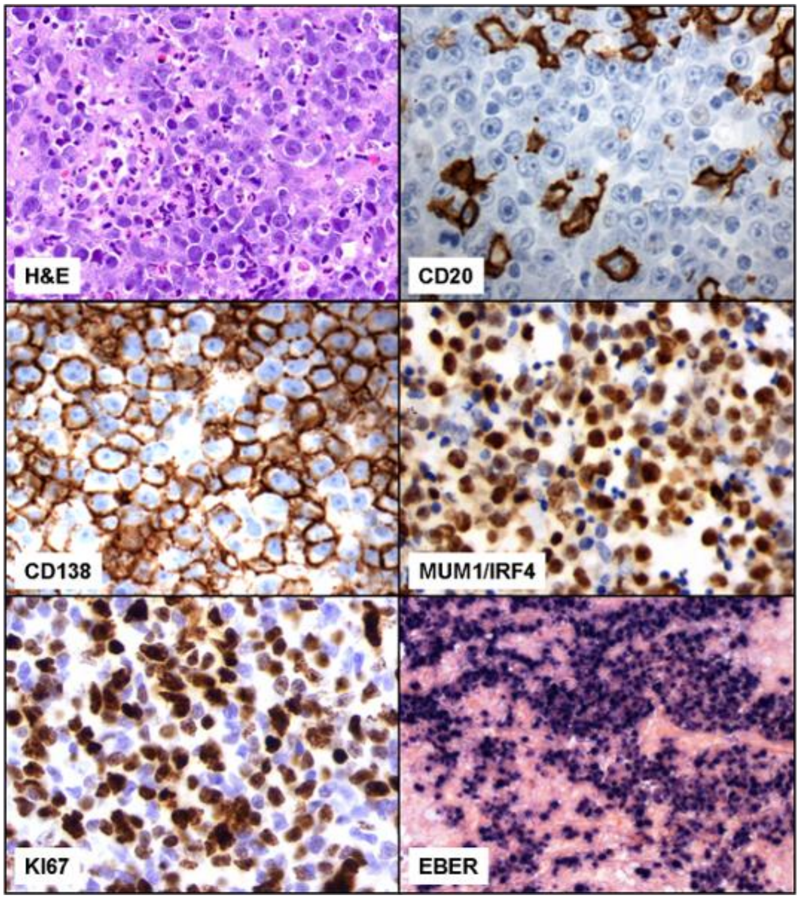

**FIGURE 1.** Representative case of PBL. In the H&E stain, a heterogeneous population of large cells with plasma cell–like appearance is shown (IHC, 100×). PBL cells are CD20 negative (IHC, 400×), CD138 (IHC, 400×), and MUM1/IRF4 positive (IHC, 400×), and show a high Ki67 expression (IHC, 400×). PBL exhibits positive EBER expression by *in situ* hybridization (ISH, 40×).

Kane and colleagues proposed minimal morphological and immunohistochemical criteria for the diagnosis of PBL of the oral cavity, which includes CD20 negativity and positivity for CD138, CD38, and/or MUM1, along with a proliferation rate based on Ki67 expression of >60% and positive EBER expression[27].

### EBER

EBER is present almost universally in the three different patterns of EBV latency, and its detection by means of fluorescent or chromogenic *in situ* hybridization (ISH) has become the standard for assessment of the presence of the EBV genome within tumor cells. In PBL, 82 and 46% of the HIV-positive and -negative cases, respectively, showed expression of EBER by ISH[5]. In a study by Vega and colleagues, expression of EBER was the only difference seen between the immunohistochemical profiles of PBL and plasmablastic PCM[46].

#### Genomic Profile

Due to the many clinicopathological similarities between PBL, DLBCL with plasmacytic differentiation, and PCM, Chang and colleagues studied the genomic profile of 16 cases of PBL, 13 cases of AIDS-related DLBCL, 13 cases of non–AIDS-related DLBCL, and eight cases of PCM utilizing an array-based comparative genomic hybridization technology[47]. Based on their findings, the genomic aberration pattern of PBL was more akin to DLBCL than PCM. This was irrespective of whether DLBCL was associated with AIDS or not. These findings suggest that PBL is best classified as a DLBCL based on genomic expression criteria. In contrast, in a series of four PBL patients, Taddesse-Heath and colleagues discovered complex cytogenetic changes that were more closely related to PCM [39]. These findings are a reflection of the likely molecularly heterogeneous nature of PBL.

#### MYC Rearrangement

The first recurrent cytogenetic abnormalities detected in PBL were translocations involving the MYC oncogene[30], a finding confirmed by others[34]. Using fluorescence ISH, Valera and colleagues examined a larger population of PBL patients and found MYC rearrangements in 49% of the cases[22]. The most common partner for the MYC oncogene was the immunoglobulin gene with translocations most often occurring in the context of complex karyotypes. Interestingly, most lymphomas, such as Burkitt lymphoma or DLBCL with MYC translocations, have a germinal-center phenotype, but PBL differs with a typical postgerminal-center phenotype. Another interesting observation was that MYC rearrangements were more often seen in EBV-positive compared to EBV-negative tumors[22]. One theory in regards to the significance of MYC translocation is that it may lead to the plasmablastic morphology and, in the process, create a more aggressive disease state[39].

### CLINICAL FEATURES

PBL has been characterized for its predilection of involving the oral cavity of HIV-positive individuals[2]. However, extraoral involvement is also frequently seen in HIV-positive cases; the most commonly affected sites are the gastrointestinal tract, lymph nodes, and skin[3]. A similar pattern is seen in patients with HIV-negative PBL, with the oral cavity and gastrointestinal tract being the most commonly involved sites[48]. However, the frequency of oral involvement is higher in HIV-positive (58%) than in HIV-negative patients (16%)[5]. Other less common extraoral sites include the CNS[2,6,49,50], paranasal sinus[6,50,51], mediastinum[6,52], lungs[24,53], liver[53,54], and testes[55]. Bone marrow involvement has been reported at 30% in both HIV-positive and -negative patients[5].

In both HIV-positive and -negative patients, 60% of patients present with an advanced clinical stage (i.e., Ann Arbor stage 3 or 4)[5]. Interestingly, in HIV-positive patients, the stage at presentation is divided into a bimodal distribution with over 80% of patients presenting with stage 1 or 4 (32 and 49%, respectively)[3]. B symptoms have been reported in 33% of HIV-positive and 50% of HIV-negative patients at diagnosis[5]. Due to scarcity of data, LDH levels, performance status, and other clinical parameters have not been appropriately evaluated in patients with PBL.

In a literature review of 112 HIV-positive PBL patients encompassing the pre- and post-HAART eras, the average CD4 count at presentation was 178 cells/mm$^3$ with an average viral load of over 86,000 copies/mL, and an average duration from HIV diagnosis to PBL diagnosis of 5 years. PBL was the initial presentation of HIV infection in approximately 5% of the cases[3]. If the advent of HAART has modified these characteristics, it is unknown at the present time.

### TREATMENT

An important aspect of the initial treatment of PBL is the use of chemotherapy. A large literature review of treated cases of PBL shows an overall response rate (ORR) to chemotherapy of 77%, with 46% of patients achieving a complete response (CR) and 31% a partial response (PR)[56]. Patients with PBL who were not treated with chemotherapy invariably died with a median survival of 3 months[5]. Cyclophosphamide, doxorubicin, vincristine, and prednisone (CHOP) and CHOP-like regimens have been used in half of the patients reported to date[56], and due to disappointing response and survival rates, the NCCN guidelines recommend against CHOP in favor of more intensive regimens, such as infusional EPOCH, HyperCVAD, or CODOX-M/IVAC[57]. However, in a recent literature review, more intensive regimens have not been shown to confer a survival advantage when compared to CHOP/CHOP-like regimens, although only a quarter of the patients reported in the literature have been treated with more intensive regimens than CHOP[56]. Certainly, the presence of MYC translocations in a proportion of patients with PBL justifies a more thorough assessment of more intensive regimens. The data on stem cell transplantation is too scant to reach any conclusions[30,38].

In patients with HIV infection and PBL, the use of HAART is recommended. In a recent study of 70 patients with HIV-positive PBL treated with chemotherapy, the use of HAART was associated with a statistical trend towards improved survival[56]. Interestingly, HAART without chemotherapy has been associated with spontaneous remissions in a few cases[58,59].

Despite a good ORR to chemotherapy, the median overall survival (OS) is 14 months with a 5-year OS rate of 31%[3]. Of note, these survival data represent a variety of therapeutic approaches in a heterogeneous patient population. Recent studies have reported the potential value of the proteasome inhibitor bortezomib in patients with DLBCL of nongerminal-center origin, which fits the profile of PBL; bortezomib blocks NF-κB, thereby sensitizing nongerminal-center lymphoma cells to cytotoxic chemotherapy[60]. To date, there are only two literature case reports on the use of bortezomib in PBL. The first case involved an HIV-positive man with PBL who started on HAART and bortezomib therapy; the patient had a rapid response after only four treatments with bortezomib but, unfortunately, succumbed to severe septic shock[35]. The second case reported resolution of PBL skin lesions while on bortezomib; this patient also died shortly after relapse[17].

Due to the lack of CD20 expression by PBL cells, the use of rituximab, a chimeric anti-CD20 monoclonal antibody, is not currently a therapeutic standard. However, it could be considered if minimal or partial expression of CD20 is detected within the malignant cells.

### PROGNOSIS

In a review of 70 HIV-positive PBL patients treated with chemotherapy, variables associated with longer survival were early clinical stage and obtaining a CR with chemotherapy[56]. Patients who obtained a CR survived longer than those patients who only achieved a PR (not reached vs. 11 months, respectively). Likewise, those with PR had a statistically significant increase in OS compared to nonresponders (11 vs. 3.5 months, respectively). In terms of subpopulation analysis, a small subset of patients carrying MYC/IgH gene rearrangement has been shown to have a very poor median OS of only 3 months. The association of MYC with poor prognosis has been suggested by additional studies[22], but based on the small sample size, it should be considered preliminary.

The use of HAART showed a trend towards statistical significance for a better survival in patients with HIV-associated PBL[56]. It is currently unclear if HIV status *per se* confers a better prognosis in patients with PBL. In a recent review of the literature, patients with PBL and HIV infection were found to have an OS of 14 months compared to 9 months in HIV-negative patients[5]. A potential explanation for this finding is that the use of HAART may restore immune surveillance to combat the tumor more efficiently. Additionally, other factors, such as extent of disease and performance status, may have also played a role in this difference. In a univariate analysis, other factors associated with a worse overall prognosis were age >60 years, advanced stage at diagnosis, bone marrow involvement, and lack of treatment with chemotherapy. In the multivariate analysis, advanced stage and no chemotherapy remained as independent factors associated with a worse prognosis[5]. The site of presentation may also affect survival. In a smaller study on 13 patients with PBL, oral presentation of PBL had a significantly increased OS compared to extraoral[36]. However, it is not clear if this simply represents a higher number of HIV-positive patients or earlier stage of disease in the population with oral involvement.

### CONCLUSION

PBL has a predilection for immunocompromised individuals based on its prevalence in both HIV-positive patients and in those undergoing solid organ transplantation. Morphologically and genomically speaking, PBL is best classified as a form of DLBCL. However, based on immunohistochemical data, PBL is much more similar to an extramedullary PCM. Clinically, it has a predilection for oral cavity involvement, a feature not typically seen in other lymphoid malignancies. Recent studies have shown a high prevalence of MYC translocations that may contribute to its aggressive nature. The pathogenesis has not clearly been defined, but is thought to involve dysregulation of terminal B-cell differentiation and apoptosis, potentially via the effects of MYC translocation and EBV infection. Treatment has been centered on CHOP chemotherapy with good response, but poor survival; however, more intensive therapies are recommended. Recent case reports of a good clinical response to bortezomib are encouraging and may provide clues to the underlying pathophysiology of PBL. Finally, it is worthwhile to highlight that no prospective therapeutic trials have been done specifically in patients with PBL.

## References

1. Stein, H., Harris, N., and Campo, E. (2008) Plasmablastic lymphoma. In WHO Classification of Tumours of the Haematopoietic and Lymphoid Tissues. Swerdlow, S., Campo, E., Harris, N., Jaffe, E., Pileri, S., Stein, H., Thile, J., and Vardiman, J., Eds. IARC, Lyon. pp. 256–257.
2. Delecluse, H.J., Anagnostopoulos, I., Dallenbach, F., Hummel, M., Marafioti, T., Schneider, U., Huhn, D., Schmidt-Westhausen, A., Reichart, P.A., Gross, U., and Stein, H. (1997) Plasmablastic lymphomas of the oral cavity: a new entity associated with the human immunodeficiency virus infection. *Blood* **89**, 1413–1420.
3. Castillo, J., Pantanowitz, L., and Dezube, B.J. (2008) HIV-associated plasmablastic lymphoma: lessons learned from 112 published cases. *Am. J. Hematol.* **83**, 804–809.
4. Rafaniello Raviele, P., Pruneri, G., and Maiorano, E. (2009) Plasmablastic lymphoma: a review. *Oral Dis.* **15**, 38–45.
5. Castillo, J.J., Winer, E.S., Stachurski, D., Perez, K., Jabbour, M., Milani, C., Colvin, G., and Butera, J.N. (2010) Clinical and pathological differences between human immunodeficiency virus-positive and human immunodeficiency virus-negative patients with plasmablastic lymphoma. *Leuk. Lymphoma* **51**, 2047–2053.
6. Colomo, L., Loong, F., Rives, S., Pittaluga, S., Martinez, A., Lopez-Guillermo, A., Ojanguren, J., Romagosa, V., Jaffe, E.S., and Campo, E. (2004) Diffuse large B-cell lymphomas with plasmablastic differentiation represent a heterogeneous group of disease entities. *Am. J. Surg. Pathol.* **28**, 736–747.
7. Kim, J.E., Kim, Y.A., Kim, W.Y., Kim, C.W., Ko, Y.H., Lee, G.K., Choi, S.J., and Jeon, Y.K. (2009) Human immunodeficiency virus-negative plasmablastic lymphoma in Korea. *Leuk. Lymphoma* **50**, 582–587.
8. Scheper, M.A., Nikitakis, N.G., Fernandes, R., Gocke, C.D., Ord, R.A., and Sauk, J.J. (2005) Oral plasmablastic lymphoma in an HIV-negative patient: a case report and review of the literature. *Oral Surg. Oral Med. Oral Pathol. Oral Radiol. Endod.* **100**, 198–206.
9. Teruya-Feldstein, J., Chiao, E., Filippa, D.A., Lin, O., Comenzo, R., Coleman, M., Portlock, C., and Noy, A. (2004) CD20-negative large-cell lymphoma with plasmablastic features: a clinically heterogenous spectrum in both HIV-positive and -negative patients. *Ann. Oncol.* **15**, 1673–1679.
10. Isaacson, P.G., Campo, E., and Harris, N.L. (2008) Large B-cell lymphoma arising in HHV8-associated multicentric Castleman disease. In WHO Classification of Tumours of Haematopoietic and Lymphoid Tissues. Swerdlow, S.H., Campo, E., Harris, N.L., Jaffe, E.S., Pileri, S., Stein, H., thiele, J., and Vardiman, J., Eds. IARC. Lyon.
11. Carbone, A. and Gloghini, A. (2008) Plasmablastic lymphoma: one or more entities? *Am. J. Hematol.* **83**, 763–764.
12. Apichai, S., Rogalska, A., Tzvetanov, I., Asma, Z., Benedetti, E., and Gaitonde, S. (2009) Multifocal cutaneous and systemic plasmablastic lymphoma in an infant with combined living donor small bowel and liver transplant. *Pediatr. Transplant.* **13**, 628–631.

13. Chabay, P., De Matteo, E., Lorenzetti, M., Gutierrez, M., Narbaitz, M., Aversa, L., and Preciado, M.V. (2009) Vulvar plasmablastic lymphoma in a HIV-positive child: a novel extraoral localisation. *J. Clin. Pathol.* **62**, 644–646.
14. Gogia, A. and Bakhshi, S. (2010) Plasmablastic lymphoma of oral cavity in a HIV-negative child. *Pediatr. Blood Cancer* **55**, 390–391.
15. Hernandez, C., Cetner, A.S., and Wiley, E.L. (2009) Cutaneous presentation of plasmablastic post-transplant lymphoproliferative disorder in a 14-month-old. *Pediatr. Dermatol.* **26**, 713–716.
16. Radhakrishnan, R., Suhas, S., Kumar, R.V., Krishnanand, G., Srinivasan, R., and Rao, N.N. (2005) Plasmablastic lymphoma of the oral cavity in an HIV-positive child. *Oral Surg. Oral Med. Oral Pathol. Oral Radiol. Endod.* **100**, 725–731.
17. Bibas, M., Grisetti, S., Alba, L., Picchi, G., Del Nonno, F., and Antinori, A. (2010) Patient with HIV-associated plasmablastic lymphoma responding to bortezomib alone and in combination with dexamethasone, gemcitabine, oxaliplatin, cytarabine, and pegfilgrastim chemotherapy and lenalidomide alone. *J. Clin. Oncol.* **28(34),** e704–708.
18. Borenstein, J., Pezzella, F., and Gatter, K.C. (2007) Plasmablastic lymphomas may occur as post-transplant lymphoproliferative disorders. *Histopathology* **51**, 774–777.
19. Brown, R.S., Campbell, C., Lishman, S.C., Spittle, M.F., and Miller, R.F. (1998) Plasmablastic lymphoma: a new subcategory of human immunodeficiency virus-related non-Hodgkin's lymphoma. *Clin. Oncol. (R. Coll. Radiol.)* **10**, 327–329.
20. Cattaneo, C., Facchetti, F., Re, A., Borlenghi, E., Majorana, A., Bardellini, E., Casari, S., Tucci, A., Conti, G., and Rossi, G. (2005) Oral cavity lymphomas in immunocompetent and human immunodeficiency virus infected patients. *Leuk. Lymphoma* **46**, 77–81.
21. Montes-Moreno, S., Gonzalez-Medina, A.R., Rodriguez-Pinilla, S.M., Maestre, L., Sanchez-Verde, L., Roncador, G., Mollejo, M., Garcia, J.F., Menarguez, J., Montalban, C., Ruiz-Marcellan, M.C., Conde, E., and Piris, M.A. (2010) Aggressive large B-cell lymphoma with plasma cell differentiation: immunohistochemical characterization of plasmablastic lymphoma and diffuse large B-cell lymphoma with partial plasmablastic phenotype. *Haematologica* **95**, 1342–1349.
22. Valera, A., Balague, O., Colomo, L., Martinez, A., Delabie, J., Taddesse-Heath, L., Jaffe, E.S., and Campo, E. (2010) IG/MYC rearrangements are the main cytogenetic alteration in plasmablastic lymphomas. *Am. J. Surg. Pathol.* **34**, 1686–1694.
23. Lin, F., Zhang, K., Quiery, A.T., Jr., Prichard, J., and Schuerch, C. (2004) Plasmablastic lymphoma of the cervical lymph nodes in a human immunodeficiency virus-negative patient: a case report and review of the literature. *Arch. Pathol. Lab. Med.* **128**, 581–584.
24. Lin, Y., Rodrigues, G.D., Turner, J.F., and Vasef, M.A. (2001) Plasmablastic lymphoma of the lung: report of a unique case and review of the literature. *Arch. Pathol. Lab. Med.* **125**, 282–285.
25. Desai, R.S., Vanaki, S.S., Puranik, R.S., Giraddi, G., and Pujari, R.V. (2007) Plasmablastic lymphoma presenting as a gingival growth in a previously undiagnosed HIV-positive patient: a case report. *J. Oral Maxillofac. Surg.* **65**, 1358–1361.
26. Gujral, S., Shet, T.M., and Kane, S.V. (2008) Morphological spectrum of AIDS-related plasmablastic lymphomas. *Indian J. Pathol. Microbiol.* **51**, 121–124.
27. Kane, S., Khurana, A., Parulkar, G., Shet, T., Prabhash, K., Nair, R., and Gujral, S. (2009) Minimum diagnostic criteria for plasmablastic lymphoma of oral/sinonasal region encountered in a tertiary cancer hospital of a developing country. *J. Oral Pathol. Med.* **38**, 138–144.
28. Chetty, R., Hlatswayo, N., Muc, R., Sabaratnam, R., and Gatter, K. (2003) Plasmablastic lymphoma in HIV+ patients: an expanding spectrum. *Histopathology* **42**, 605–609.
29. Goedhals, J., Beukes, C.A., and Cooper, S. (2006) The ultrastructural features of plasmablastic lymphoma. *Ultrastruct. Pathol.* **30**, 427–433.
30. Dawson, M.A., Schwarer, A.P., McLean, C., Oei, P., Campbell, L.J., Wright, E., Shortt, J., and Street, A.M. (2007) AIDS-related plasmablastic lymphoma of the oral cavity associated with an IGH/MYC translocation--treatment with autologous stem-cell transplantation in a patient with severe haemophilia-A. *Haematologica* **92**, e11–12.
31. Valenzuela, A.A., Walker, N.J., and Sullivan, T.J. (2008) Plasmablastic lymphoma in the orbit: case report. *Orbit* **27**, 227–229.
32. Ferrazzo, K.L., Mesquita, R.A., Aburad, A.T., Nunes, F.D., and de Sousa, S.O. (2007) EBV detection in HIV-related oral plasmablastic lymphoma. *Oral Dis.* **13**, 564–569.
33. Ortega, K.L., Arzate-Mora, N., Ceballos, S., and Martin-Rico, P. (2007) Images in HIV/AIDS. Oral plasmablastic lymphoma. *AIDS Read.* **17**, 446–447.
34. Bogusz, A.M., Seegmiller, A.C., Garcia, R., Shang, P., Ashfaq, R., and Chen, W. (2009) Plasmablastic lymphomas with MYC/IgH rearrangement: report of three cases and review of the literature. *Am. J. Clin. Pathol.* **132**, 597–605.
35. Bose, P., Thompson, C., Gandhi, D., Ghabach, B., and Ozer, H. (2009) AIDS-related plasmablastic lymphoma with dramatic, early response to bortezomib. *Eur. J. Haematol.* **82**, 490–492.
36. Hansra, D., Montague, N., Stefanovic, A., Akunyili, I., Harzand, A., Natkunam, Y., de la Ossa, M., Byrne, G.E., and Lossos, I.S. (2010) Oral and extraoral plasmablastic lymphoma: similarities and differences in clinicopathologic characteristics. *Am. J. Clin. Pathol.* **134**, 710–719.

37. Reid-Nicholson, M., Kavuri, S., Ustun, C., Crawford, J., Nayak-Kapoor, A., and Ramalingam, P. (2008) Plasmablastic lymphoma: cytologic findings in 5 cases with unusual presentation. *Cancer* **114**, 333–341.
38. Simonitsch-Klupp, I., Hauser, I., Ott, G., Drach, J., Ackermann, J., Kaufmann, J., Weltermann, A., Greinix, H.T., Skrabs, C., Dittrich, C., Lutz, D., Potter, R., Mannhalter, C., Lechner, K., Chott, A., and Jaeger, U. (2004) Diffuse large B-cell lymphomas with plasmablastic/plasmacytoid features are associated with TP53 deletions and poor clinical outcome. *Leukemia* **18**, 146–155.
39. Taddesse-Heath, L., Meloni-Ehrig, A., Scheerle, J., Kelly, J.C., and Jaffe, E.S. (2010) Plasmablastic lymphoma with MYC translocation: evidence for a common pathway in the generation of plasmablastic features. *Mod. Pathol.* **23**, 991–999.
40. Carbone, A., Cesarman, E., Spina, M., Gloghini, A., and Schulz, T.F. (2009) HIV-associated lymphomas and gamma-herpesviruses. *Blood* **113**, 1213–1224.
41. Cioc, A.M., Allen, C., Kalmar, J.R., Suster, S., Baiocchi, R., and Nuovo, G.J. (2004) Oral plasmablastic lymphomas in AIDS patients are associated with human herpesvirus 8. *Am. J. Surg. Pathol.* **28**, 41–46.
42. Verma, S., Nuovo, G.J., Porcu, P., Baiocchi, R.A., Crowson, A.N., and Magro, C.M. (2005) Epstein-Barr virus- and human herpesvirus 8-associated primary cutaneous plasmablastic lymphoma in the setting of renal transplantation. *J. Cutan. Pathol.* **32**, 35–39.
43. Brown, R.S., Power, D.A., Spittle, H.F., and Lankester, K.J. (2000) Absence of immunohistochemical evidence for human herpesvirus 8 (HHV8) in oral cavity plasmablastic lymphoma in an HIV-positive man. *Clin. Oncol. (R. Coll. Radiol.)* **12**, 194.
44. Folk, G.S., Abbondanzo, S.L., Childers, E.L., and Foss, R.D. (2006) Plasmablastic lymphoma: a clinicopathologic correlation. *Ann. Diagn. Pathol.* **10**, 8–12.
45. Schebesta, M., Heavey, B., and Busslinger, M. (2002) Transcriptional control of B-cell development. *Curr. Opin. Immunol.* **14**, 216–223.
46. Vega, F., Chang, C.C., Medeiros, L.J., Udden, M.M., Cho-Vega, J.H., Lau, C.C., Finch, C.J., Vilchez, R.A., McGregor, D., and Jorgensen, J.L. (2005) Plasmablastic lymphomas and plasmablastic plasma cell myelomas have nearly identical immunophenotypic profiles. *Mod. Pathol.* **18**, 806–815.
47. Chang, C.C., Zhou, X., Taylor, J.J., Huang, W.T., Ren, X., Monzon, F., Feng, Y., Rao, P.H., Lu, X.Y., Fabio, F., Hilsenbeck, S., Creighton, C.J., Jaffe, E.S., and Lau, C.C. (2009) Genomic profiling of plasmablastic lymphoma using array comparative genomic hybridization (aCGH): revealing significant overlapping genomic lesions with diffuse large B-cell lymphoma. *J. Hematol. Oncol.* **2**, 47.
48. Castillo, J.J., Winer, E.S., Stachurski, D., Perez, K., Jabbour, M., Milani, C., Colvin, G., and Butera, J.N. (2011) HIV-negative plasmablastic lymphoma: not in the mouth. *Clin. Lymphoma Myeloma Leuk.*, in press.
49. Shuangshoti, S., Assanasen, T., Lerdlum, S., Srikijvilaikul, T., Intragumtornchai, T., and Thorner, P.S. (2008) Primary central nervous system plasmablastic lymphoma in AIDS. *Neuropathol. Appl. Neurobiol.* **34**, 245–247.
50. Ustun, C., Reid-Nicholson, M., Nayak-Kapoor, A., Jones-Crawford, J., McDonald, K., Jillella, A.P., and Ramalingam, P. (2009) Plasmablastic lymphoma: CNS involvement, coexistence of other malignancies, possible viral etiology, and dismal outcome. *Ann. Hematol.* **88**, 351–358.
51. Vega, F., Lin, P., and Medeiros, L.J. (2005) Extranodal lymphomas of the head and neck. *Ann. Diagn. Pathol.* **9**, 340–350.
52. Nasta, S.D., Carrum, G.M., Shahab, I., Hanania, N.A., and Udden, M.M. (2002) Regression of a plasmablastic lymphoma in a patient with HIV on highly active antiretroviral therapy. *Leuk. Lymphoma* **43**, 423–426.
53. Sarode, S.C., Zarkar, G.A., Desai, R.S., Sabane, V.S., and Kulkarni, M.A. (2009) Plasmablastic lymphoma of the oral cavity in an HIV-positive patient: a case report and review of literature. *Int. J. Oral Maxillofac. Surg.* **38**, 993–999.
54. Riedel, D.J., Gonzalez-Cuyar, L.F., Zhao, X.F., Redfield, R.R., and Gilliam, B.L. (2008) Plasmablastic lymphoma of the oral cavity: a rapidly progressive lymphoma associated with HIV infection. *Lancet Infect. Dis.* **8**, 261–267.
55. Dong, H.Y., Scadden, D.T., de Leval, L., Tang, Z., Isaacson, P.G., and Harris, N.L. (2005) Plasmablastic lymphoma in HIV-positive patients: an aggressive Epstein-Barr virus-associated extramedullary plasmacytic neoplasm. *Am. J. Surg. Pathol.* **29**, 1633–1641.
56. Castillo, J.J., Winer, E.S., Stachurski, D., Perez, K., Jabbour, M., Milani, C., Colvin, G., and Butera, J.N. (2010) Prognostic factors in chemotherapy-treated patients with HIV-associated plasmablastic lymphoma. *Oncologist* **15**, 293–299.
57. NCCN Practice Guidelines in Oncology. AIDS-related B-cell lymphomas (AIDS-2). Available at http://www.nccn.org/professionals/physician_gls/PDF/nhl.pdf. Accessed on November 27, 2010.
58. Armstrong, R., Bradrick, J., and Liu, Y.C. (2007) Spontaneous regression of an HIV-associated plasmablastic lymphoma in the oral cavity: a case report. *J. Oral Maxillofac. Surg.* **65**, 1361–1364.
59. Gilaberte, M., Gallardo, F., Bellosillo, B., Saballs, P., Barranco, C., Serrano, S., and Pujol, R.M. (2005) Recurrent and self-healing cutaneous monoclonal plasmablastic infiltrates in a patient with AIDS and Kaposi sarcoma. *Br. J. Dermatol.* **153**, 828–832.
60. Dunleavy, K., Pittaluga, S., Czuczman, M.S., Dave, S.S., Wright, G., Grant, N., Shovlin, M., Jaffe, E.S., Janik, J.E., Staudt, L.M., and Wilson, W.H. (2009) Differential efficacy of bortezomib plus chemotherapy within molecular subtypes of diffuse large B-cell lymphoma. *Blood* **113**, 6069–6076.
